# Olfactory interference on the emotional processing speed of visual stimuli: The influence of facial expressions intensities

**DOI:** 10.1371/journal.pone.0264261

**Published:** 2022-05-17

**Authors:** Matheus Henrique Ferreira, Patricia Renovato Tobo, Carla Regina Barrichello, Mirella Gualtieri

**Affiliations:** 1 Department of experimental Psychology, Instituto de Psicologia, Universidade de São Paulo, São Paulo, Brazil; 2 Natura Cosméticos S.A., São Paulo, Brazil; Universita degli Studi di Pisa, ITALY

## Abstract

Research on olfactory stimulation indicates that it can influence human cognition and behavior, as in the perception of facial expressions. Odors can facilitate or impair the identification of facial expressions, and apparently its hedonic valence plays an important role. However, it was also demonstrated that the presentation of happiness and disgust faces can influence the emotional appraisal of odorants, indicating a bilateral influence in this phenomenon. Hence, it’s possible that odor influences on emotional categorization vary depending on the intensity of expressions. To investigate this hypothesis, we performed an emotion recognition task using facial expressions of five emotional categories (happiness, fear, disgust, anger and sadness) with ten different intensities. Thirty-five participants completed four blocks of the task, each with a different olfactory condition, and we found that odorants’ effects varied according to the facial expressions intensity. Odorants enhanced the Reaction Time (RT) differences between threshold and high-intensity expressions for disgust and fear faces. Also, analysis of the RT means for high-intensity facial expressions revealed that the well-known advantage in recognition of happiness facial expressions, compared to other emotions, was enhanced in the positive olfactory stimulation and decreased in the negative condition. We conclude that olfactory influences on emotional processing of facial expressions vary along intensities of the latter, and the discrepancies of past research in this field may be a result of a bilateral effect in which the odorants influence the identification of emotional faces just as the facial expressions influence the emotional reaction to the odor.

## Introduction

Anatomical and physiological features of the olfactory system make it different from other sensory modalities and especially linked with emotional processing, since its neuroanatomy is reciprocally interlaced with primary emotional areas [1]. Emotional responses to olfactory stimuli are associated to objective autonomic measures, such as heart rate, skin conductance, and facial musculature activity [2,3]. Studies have found that unpleasant odorants can increase heart rate [2–6], and stimuli to which individuals attribute high arousal (both pleasant and unpleasant) are associated with increased skin conductance responses, compared to odorants with low arousal [6,7]. As to cognitive implications, odors can influence the emotional processing of stimuli in other modalities, such as facial expressions [8–12]. As described by Marcel Proust in his book “In search of lost time”, odor stimuli presentation is capable of eliciting involuntary and vivid memories. This is an example of the powerful connections between olfaction and emotional memory, probably due to anatomical proximity with the limbic system [1].

Research on olfactory function have also demonstrated that it has implications on mental health disturbances. For example, schizophrenia patients have difficulty in odorant identification and lower olfactory sensibility. This could be linked to the connection between olfaction and emotion, or simply because of the social and cognitive deficit presented in this condition [13–15]. Clinical depression also might be linked to changes in odorant perception, [16,17]. Kohli et al demonstrated that not just depressive patients had a lower odor identification, but also individuals with olfactory deficits presented depressive symptoms [17]. For some other neural disorders the findings are somewhat controversial, since subjects diagnosed with Autism Spectrum Disorder frequently present impairment of odor recognition [18]. These researches demonstrate the importance of odor-induced affective states and its intricate relation to different aspects of social interaction, in the present paper we considered the recognition of low-intensity facial expressions as a means to study the effects of odour-induced affective states.

Here we sought to bring further contributions to this topic, since previous experiments on identification of facial expressions concomitant with olfactory stimulation show heterogeneous results and are inconclusive in describing how the hedonic valence (pleasantness) of a smell interferes with the recognition of the emotion expressed. For instance, our study indicated that valence-congruent effects might be modulated by the intensity of the affective reaction elicited by the stimuli in both modalities, olfactory and visual, since facial expressions also influence the emotional state of the individuals. Leppänen and Hietanen demonstrated that reaction times (RTs) for identifying a facial expression were significantly shorter when happiness faces were paired with a pleasant odor, compared to unpleasant or neutral, but no differences were found for disgust faces [12]. Similarly, Leleu et al. showed that the covert presence of a pleasant odor (i.e., strawberry) lowered the identification threshold for subtle facial expressions of happiness, and an unpleasant odor (butyric acid) improved the detection of expressions of disgust and anger [8]. However, a third study found that expressions of disgust were identified faster in the presence of an olfactory stimuli, independently of their subjective valence; and expressions of happiness required longer RTs to be recognized, regardless of odor stimulation [19]. These results suggest that both the positive and negative olfactory stimuli facilitated the perception of disgusted faces and no differences were found for happiness expressions. Also, Leleu et al. states that olfactory stimulation can facilitate the identification of low-intensity facial expressions of specific categories of emotion which meaning are congruent to the olfactory context, such as disgust and happiness [8].

It is important to note that other factors can affect the odor influences on face perception. For example, positive olfactory stimulation can improve the visual attention to happiness expressions, but this effect decreases with time [20]. Also, Cook et al. showed that facial expressions presentation influences the emotional appraisal of odorants [9]. This is an evidence of a bilateral influence between the olfactory and face stimuli. Apparently, there are three important factors modulating the olfactory influences on facial expressions identification. First, the congruency of the hedonic valence between the odorant and the facial expression [8,12]. Since olfactory stimulation per se can induce some basic emotions, such as disgust, but not others, such as sadness, the emotional category of the facial expression can also be considered as a significant factor [8]. Lastly, the study of Cook et al. indicates a bilateral effect in this interaction, demonstrating that facial expressions presentation can also influence subject’s emotional appraisal of odorants [9]. Considering that face stimuli influence the emotional response to odorants, we hypothesized that olfactory stimulation effects in facial expressions recognition could vary for different intensities of facial expressions. To test this hypothesis, we created a matching to sample task using the method described in Leleu et al. [8] and measured the RT for different odor conditions.

## Materials and methods

### Participants

We based our sample size on similar experiments conducted previously. Sample sizes in these studies were almost fifty participants, but splitted in groups of less than 25 subjects to conduct the analysis [8,12,19]. Other experiments using similar experimental paradigm have also used a sample smaller than twenty-five subjects [21,22]. Our sample was composed by thirty-five participants (15 men and 20 women, mean age = 32.4 ± 9.8 years, range: 20–53 years). All subjects provided written informed consent before the experiment. The study protocol was approved by the Human Research Ethics Committee, Institute of Psychology, University of São Paulo (CEPH-IPUSP; CAAE 80319317.3.0000.5561), Brazil, and it complies with the Declaration of Helsinki for Medical Research involving Human Subjects [23].

### Odor stimuli

The odor stimuli were butyric acid, isoamyl acetate (both from Sigma-Aldrich, Munich, Germany) and lemongrass essence (Fazenda Alpina, São Paulo, Brazil). The control, or neutral, condition consisted of no odorant presentation. Both butyric acid and isoamyl acetate were diluted in mineral oil at a concentration of 10^−5^ v/v and 10^−2^ v/v, respectively. The lemongrass essence was used at a 30% dilution, also in mineral oil.

### Visual stimuli

**Fig 1:**
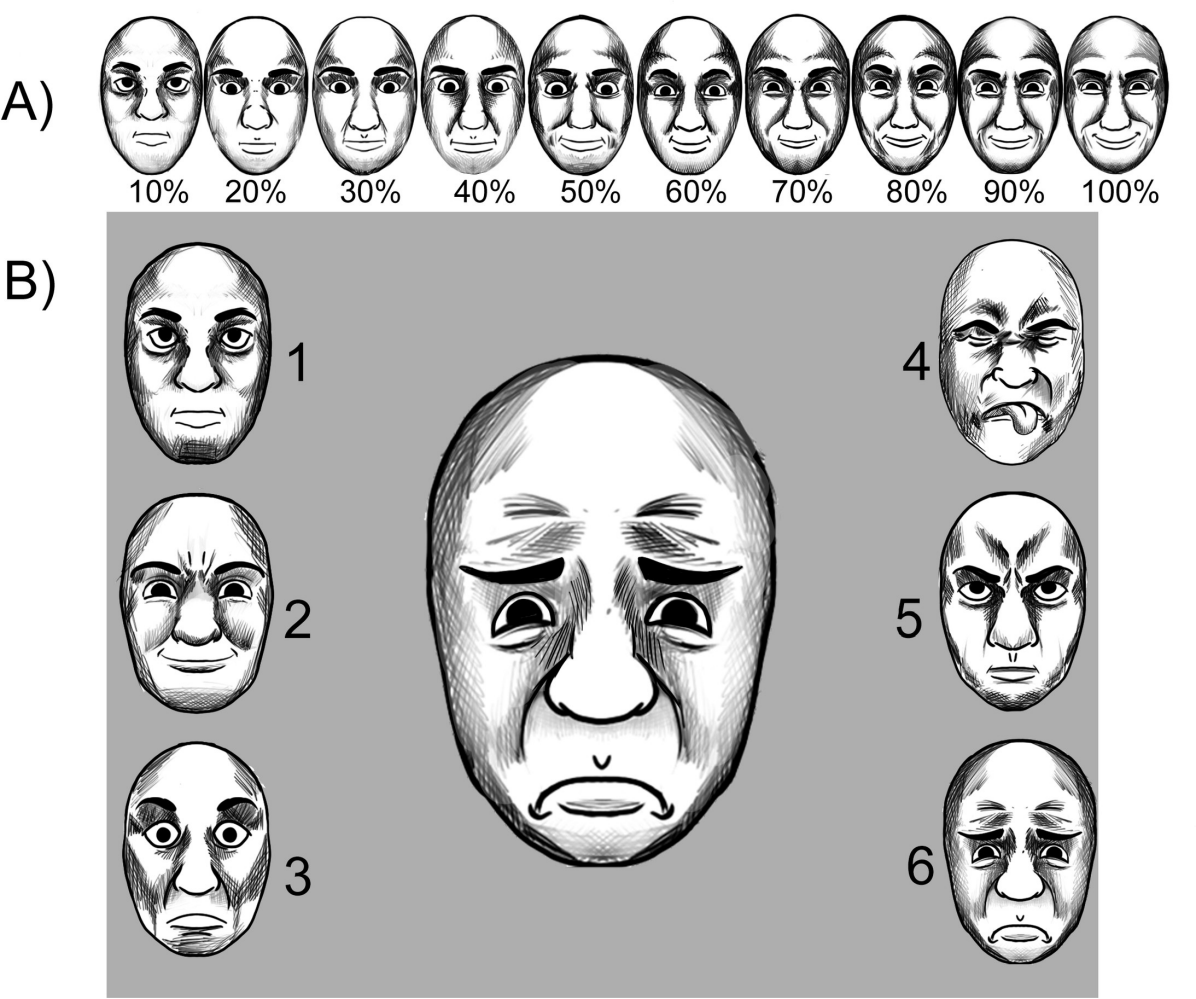
Visual stimuli examples. (A) Illustrative example of the linear continuum of morphs created by morphing the neutral expression with the happiness face. (B) Illustrative example of the facial identification task. The large expression in the center is a sadness expression. These images do not correspond to the actual stimuli used in the experiment, and were created just for the purpose of illustrating the facial morphing procedure and the facial identification task in this article. The images used in the experiment (obtained from The NimStim set of facial expressions) are protected by copyrights and not freely available to be published.

We used the images of four models (2, 3, 21, and 36) from the NimStim set [24] who presented facial expressions of happiness, sadness, disgust, fear, anger, and neutral. To obtain images with low-intensity expressions, we followed the protocol described by Leleu et al. to generate an incremental gradient of intensities [8]. Each emotional expression was morphed with the neutral face, resulting in a gradient of 10 images with varying intensity of facial expressions from 10% to 100% (Fig 1A). A total of 51 images of each model were obtained (10 images of each emotion plus the neutral expression). The images were morphed using FaceMorpher software (Luxand, Rockville, MD, USA). As in Leleu et al. [8], we conducted a pilot study (n = 5) with members from our laboratory team to equalize the intensity between emotions (2 women; mean age = 33.4; range: 25–38). The objective was to measure the lowest intensity in which all participants correctly identified each expression category and create a new linear continuum set in which all emotions were equal (in terms of intensity).

In each trial, the target stimulus was presented in the center of the screen, together with six smaller 100% intensity faces that were displayed as references for the judgments on the left side (neutral, happiness, and fear) and right side (disgust, anger, and sadness). All of the images were presented on a gray background (Fig 1B).

### Procedure

In a match-to-sample task, the subjects were instructed to look at a fixation cross that was presented for 2000 ms and then indicate the emotion they judged was expressed in the target image (facial expression) by selecting one of the six numbered options using a numeric keyboard. We modified the keyboard, following Schubert, Hagemann, Voss, Schankin, and Bergmann [25], so the position of the keys would match the positions of the numbers that were assigned to the faces on the screen. The target image only disappeared after the subject chose one of the matching facial expressions. The stimuli were presented and the participants’ responses were recorded using E-Prime 2.0 software (Psychology Software Tools, Sharpsburg, PA, USA).

**Fig 2:**
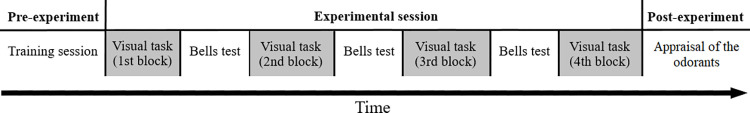
Timeline of the experimental procedure. Timeline showing the procedures conducted in pre-experiment, experimental session and post-experiment moments. The stages of the experimental session in which an odor condition was present (butyric acid, isoamyl acetate, lemongrass or no odor) are marked in grey.

The participants sat approximately 60 cm from the computer screen and were instructed to respond as soon as they identified the emotional expression. The full experimental session comprised three steps: (1) training, (2) four-block experimental session, and (3) subjective appraisal of the odorants (see Fig 2). The training session included 16 images with the neutral and most intense facial expressions of each emotion (80%, 90%, and 100%). The subjects had to correctly identify at least 13 of the 16 expressions that were presented before the experiment began, and no odor stimulus was presented during training. All subjects were able to correctly identify the minimum number of facial expressions during this training session. The experimental session was divided into four blocks with 51 images from the same model (i.e., 10 intensity levels of each of the basic emotions and the neutral face). The four blocks of trials differed according to the odor condition that was associated with it (butyric acid, isoamyl acetate, lemongrass and no odor). Olfactory stimuli presentation was conducted by applying the odorant into the foam cover of the microphone attached to the headphones [8]. Each odorant was applied in a different headset, and clean (no odorant) headphones were used for the control condition. All odors were absorbed into the microphone immediately before its presentation, between task blocks. The volume of the substance that was applied was 50 μl (one drop) isoamyl acetate and 100 μl (two drops) for butyric acid and lemongrass. The volume of each substance to be applied in the microphone were defined in a pilot study (*n* = 6), conducted to equalize the intensity of the odors, in which participants rated the intensity of the three odorants after they were applied to the microphone. We used the volume that was established for butyric acid (100 μl) by Leleu et al. [8] as a baseline for this pilot study.

Between each block, the subjects performed the Bells test, in which they were requested to mark as many bells as possible on a paper sheet [26]. Odors presentation order was pseudo-randomized using an online website (www.random.org). After the four blocks of trials in the experiment, the experimenter informed the participants about the presence of odorants during the experimental session and requested them to rate the odorants in terms of valence (pleasant or unpleasant). We then organized the data according to the subject`s appraisal of the odorant, for example, the pleasant condition corresponded to the odorant that the participant considered as pleasant. In cases which more than one odorant were considered as pleasant/unpleasant, the odor chosen would be the one that the majority of subjects rated with the same valence. After the procedure, the foam cover of the microphone in each headset was replaced for a new one, in order to avoid fluctuations of odorant concentration between participants.

### Data analysis

As described above, we organized the data according to the valence that each subject attributed to the odorants. The final dataset was composed of three conditions, unpleasant, pleasant and neutral. We first verified the *threshold for correct identification* of each emotion, by computing the lowest intensity in which the participants correctly identified the facial expression. Then we tested the null hypothesis of no difference among thresholds of each emotion by means of a one-way ANOVA with Emotion as within subjects factor. In cases where the subject presented a correct response for a determined intensity but committed errors for expressions of higher intensity, the threshold was established by calculating the mean value between the threshold intensity and the next intensity in which a correct response was presented [8]. For example, if the participant correctly identified the facial expressions with 40% and 60% of intensity but was not able to detect the expression of 50%, the threshold for perception would be (40% + 60%)/2 = 50%. In the second step of the analysis we established two measures to analyze the influence of the olfactory stimulation in subject’s Reaction Time (RT), which were the *Reaction Time for different facial expressions intensities* and the *Minimum Reaction Time for high-intensity facial expressions*. A one-way ANOVA was performed using each expression intensity (from 10 to 100) of all emotions to test the null hypothesis of no difference among RT of each facial expressions intensities. The *Reaction Time for different facial expressions intensities* was established based on the results of the threshold analysis. We organized the data according to three different intensities, the threshold, medium and full expressions. Threshold expressions corresponded to the intensity nearest the mean threshold of all emotions. Conversely, the full category was composed by the photographs with highest expression intensity, represented by 100, and medium facial expressions would be the intensity in the middle of threshold and full expressions. For this measure, we performed a three-way ANOVA with Odor, Emotion and Intensity as within subjects factors. The second measure used to analyze the RT consisted of computing the *minimum Reaction Time values for the most intense emotions* (80, 90, and 100) in each olfactory stimulation conditions. Using these data, we also performed two-way ANOVAs, with odor and expression as within-subjects factors for testing the null hypothesis of no difference among minimum RT means of the emotion categories. Effect sizes were measured using partial-eta squared (η_p_^2^), and for post hoc analysis we conducted Bonferroni correction. SPSS 23.0 software (SPSS, Chicago, IL, USA) was used to perform the statistical analyses. Values of *p* < .05 were considered statistically significant.

## Results

First, we organized all data (both Reaction Time and threshold for correct perception) in three different conditions (pleasant, unpleasant and no odor), according to each subject’s appraisal of the odorants.

### Threshold for identification of emotion

**Fig 3:**
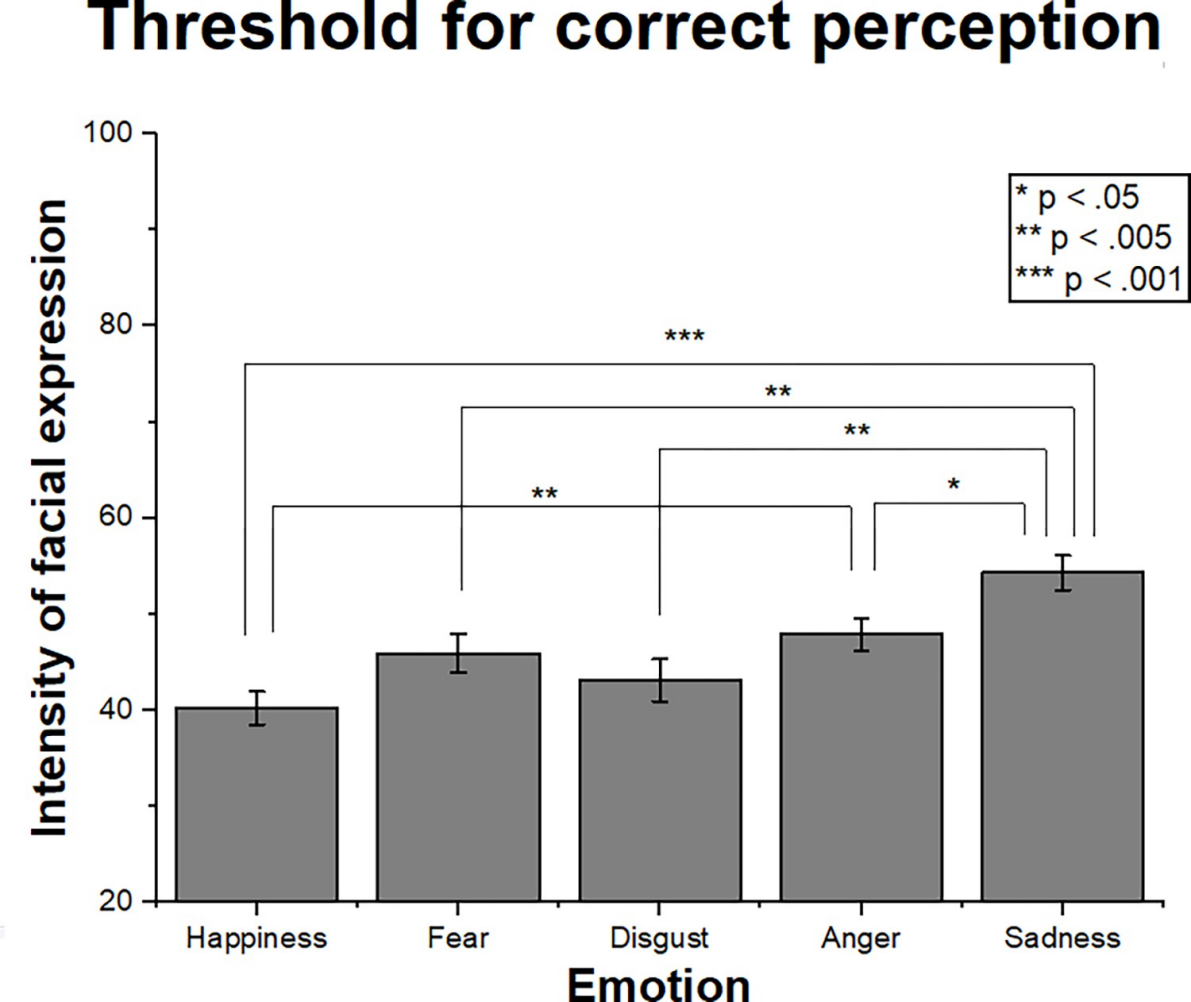
Perception thresholds of each emotion category. Mean intensities and standard errors for correct identification of each emotion for all odor conditions grouped. Sadness presented the highest perception threshold between the emotions, and fear was significantly higher than happiness. The significant differences are illustrated by asterisk brackets.

The Emotion factor was significant (F = 10.69; p < .001; η_p_^2^ = .24). Fig 3 shows the means and standard errors of threshold for each emotion with all odors grouped. Sadness presented a higher threshold (54.3) than all other emotions (from 40.2 to 47.9; p < .05), and happiness (40.2) was significantly lower than anger (47.9; p < .005) in this measure. The differences between the other emotions were not significant. The mean threshold of all emotions grouped was 46.3 (SE 1.88), which means that the 50-intensity expressions are the best to represent a threshold intensity between the stimuli presented.

### Reaction time for different facial expressions intensities

**Fig 4:**
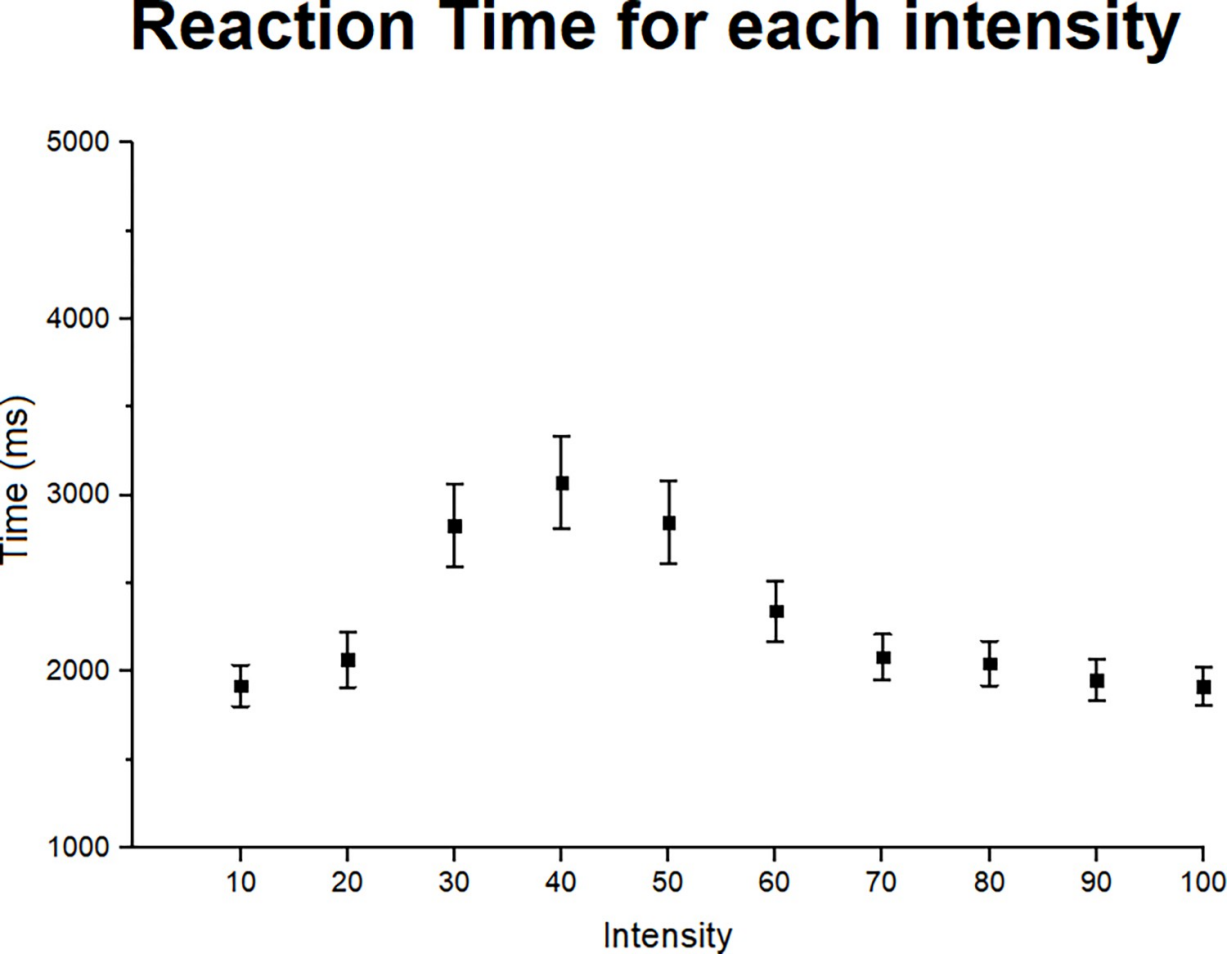
RT for each intensity. RT means and standard errors for each intensity with all emotions and odor conditions grouped.

The means and standard errors of RT in each intensity for all emotions are illustrated in Fig 4. The Intensity factor was significant (F = 15.49; p < 001; η_p_^2^ = .31) as the 10 and 100 intensities led to the lowest and highest RT means, respectively. For low intensities, (10 to 40) the RT means increased along with the facial expression intensity, forming an ascending curve, until it reaches the threshold level and the values start to decrease. in other words, after threshold level of a facial expression intensity is reached the RT tends to decrease resulting in a descending curve.

We first established the threshold, medium and full intensities of facial expressions. Given that 50 was the intensity nearest the mean threshold of all emotions grouped (46.3), it was considered as the threshold intensity. Since there were no facial expressions which corresponded to the middle intensity between threshold (50) and full intensities (100), we decided to use 70 as medium intensity because it showed greater RT difference to the 100, as shown by Fig 4, compared to 80 intensity.

**Fig 5:**
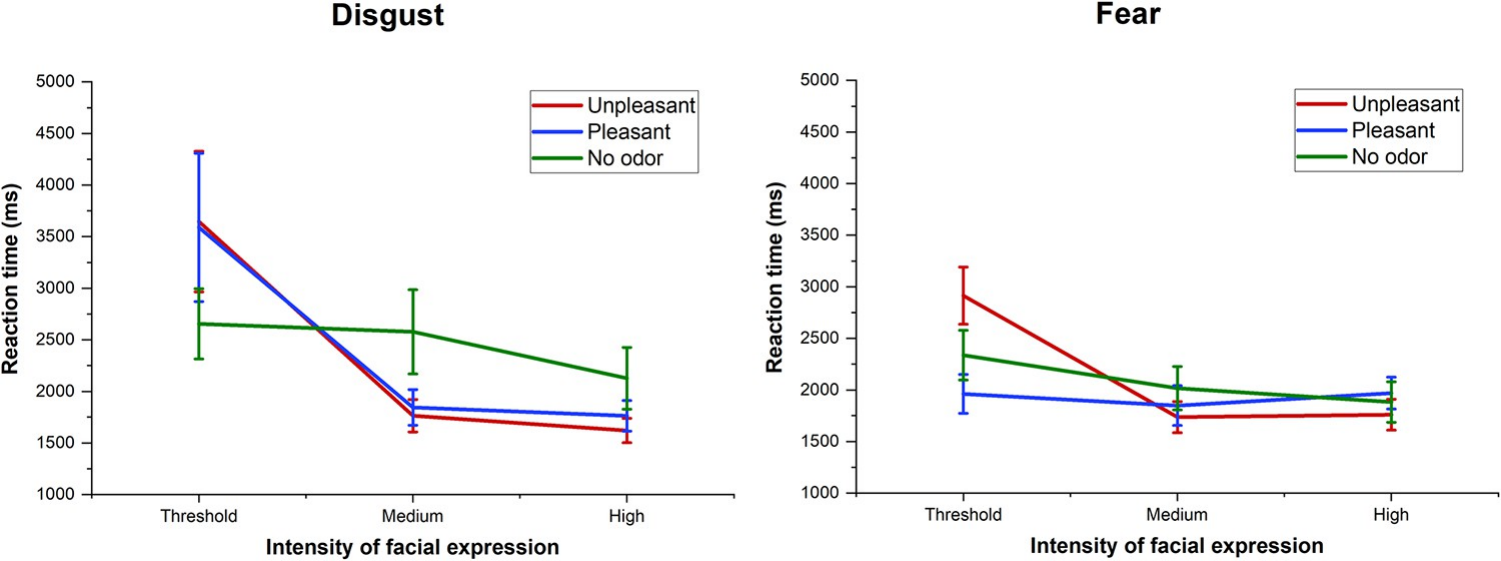
Analysis of RT between intensities for disgust and fear. Graphs showing RT means and standard errors for disgust and fear at threshold, medium and high expression intensities in different odor conditions.

Results of the three-way ANOVA, using Odor, Emotion and Intensity (50, 70 and 100) as factors within subjects showed that the interaction between the three factors (Odor vs Emotion vs. Intensity) was significant (F = 1.68; p < .05; η_p_^2^ = .04). Also, the interaction between factors Emotion vs. Intensity (F = 2.13; p < .05; η_p_^2^ = .05) was also significant. This demonstrates that the RT means varied according to the emotion and intensity of the expression. The 50 intensity was significantly lower than others (both p < .001), and there was also a significant distance between 70 and 100 (p < .05). Hence, the highest difference in RT means was between 50 and 70 intensities. Emotion (F = 4.73; p < .005; η_p_^2^ = .12) and Intensity (F = 25.59; p < .001; η_p_^2^ = .42) factors reached statistical significance, but the Odor factor (F < 1) and the interactions between Odor vs. Intensity and Odor vs. Emotion were not. The one-way ANOVAs for each emotion revealed that the descending curve of RT means along intensities (threshold, medium and full) were significantly different between olfactory conditions for the disgust and fear expressions, but not for the other emotional categories. For disgust expressions (Fig 5), both pleasant and unpleasant conditions enhanced the angle of the descending curve across intensities. The difference between the highest and lowest RT mean along intensities for disgust expressions was 2025.6 ms for the unpleasant condition and 1826.6 ms in the pleasant. In contrast, the no odor condition only presented a 528.3 ms difference between the intensities. The unpleasant condition significantly enhanced the angle of the curve for fear expressions (Fig 5), presenting a mean difference of 1154.2 ms between intensities, but not in the pleasant and no odorant conditions (113 ms and 454 ms, respectively). S1 Fig illustrates the mean RT differences and standard errors between the intensities of happiness, anger and sadness expressions in each odor condition. Although results did not present any significant differences for happiness, it is interesting to note that it was the only emotional category which presented the lower RT means in the pleasant condition for all intensities analyzed. Also, the highest difference in RT means between the intensities was found in the pleasant condition (727.3 ms), (459.6 ms for unpleasant and 708.1 ms for no odor condition).

### Minimum reaction time for high-intensity facial expressions (80, 90 and 100)

**Fig 6:**
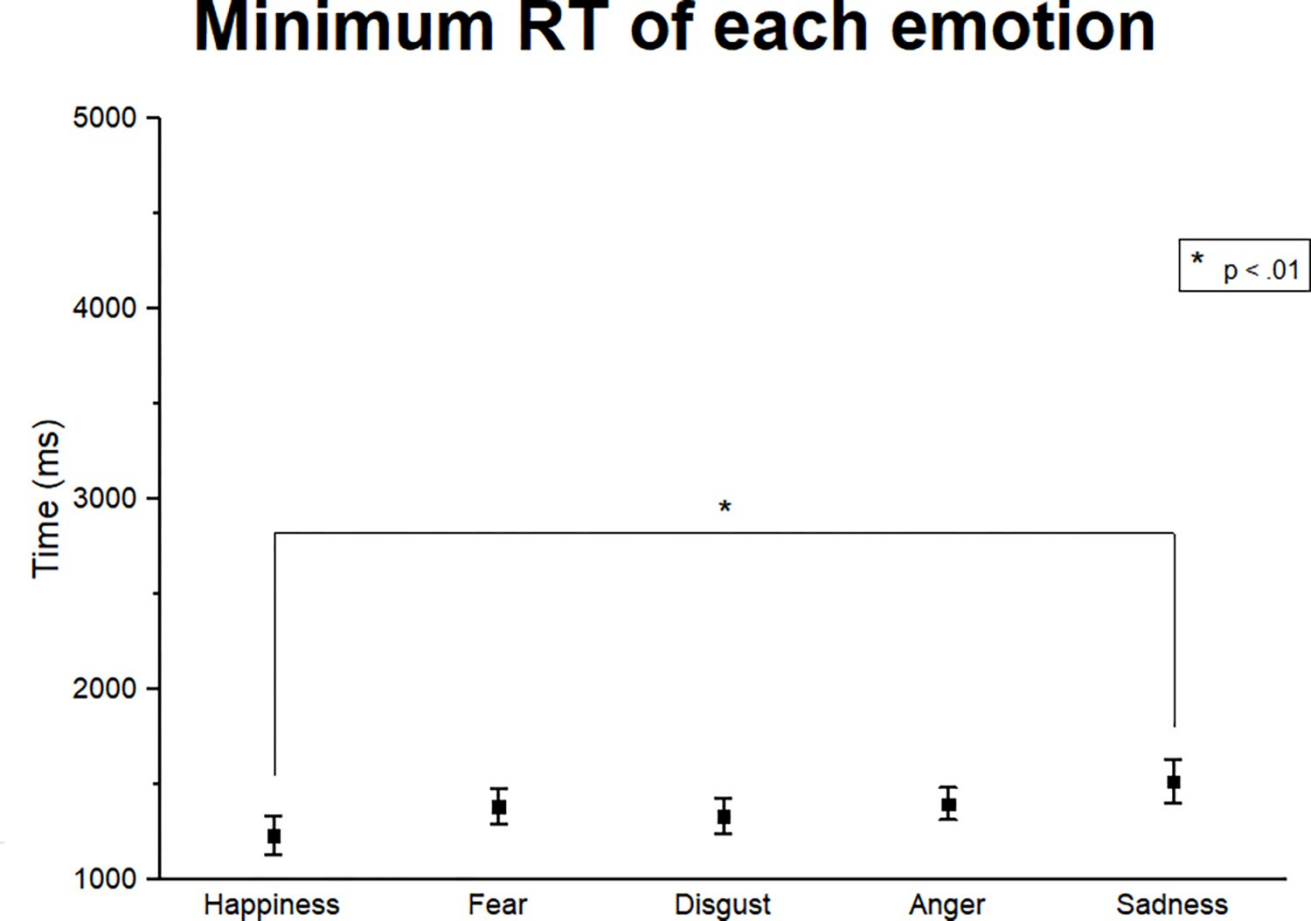
Minimum RT of each emotion for high-intensity facial expressions (80, 90 and 100). Means and standard errors of the minimum RT of each emotion for all odor conditions grouped. Happiness was recognized faster than sadness expressions (p < .005), but no other differences between emotions were observed. Significant differences are illustrated by asterisk brackets.

To compare the time for identification of high-intensity expressions, we computed the minimum RT value between 80, 90 and 100 intensities of each emotion in each condition. As illustrated by Fig 6, sadness and happiness significantly differed in this measure. Since the mean values of minimum RT for all emotions were significantly below the 2000 ms, responses greater than 3000 ms were considered as outliers and excluded from the next step of this analysis.

**Fig 7:**
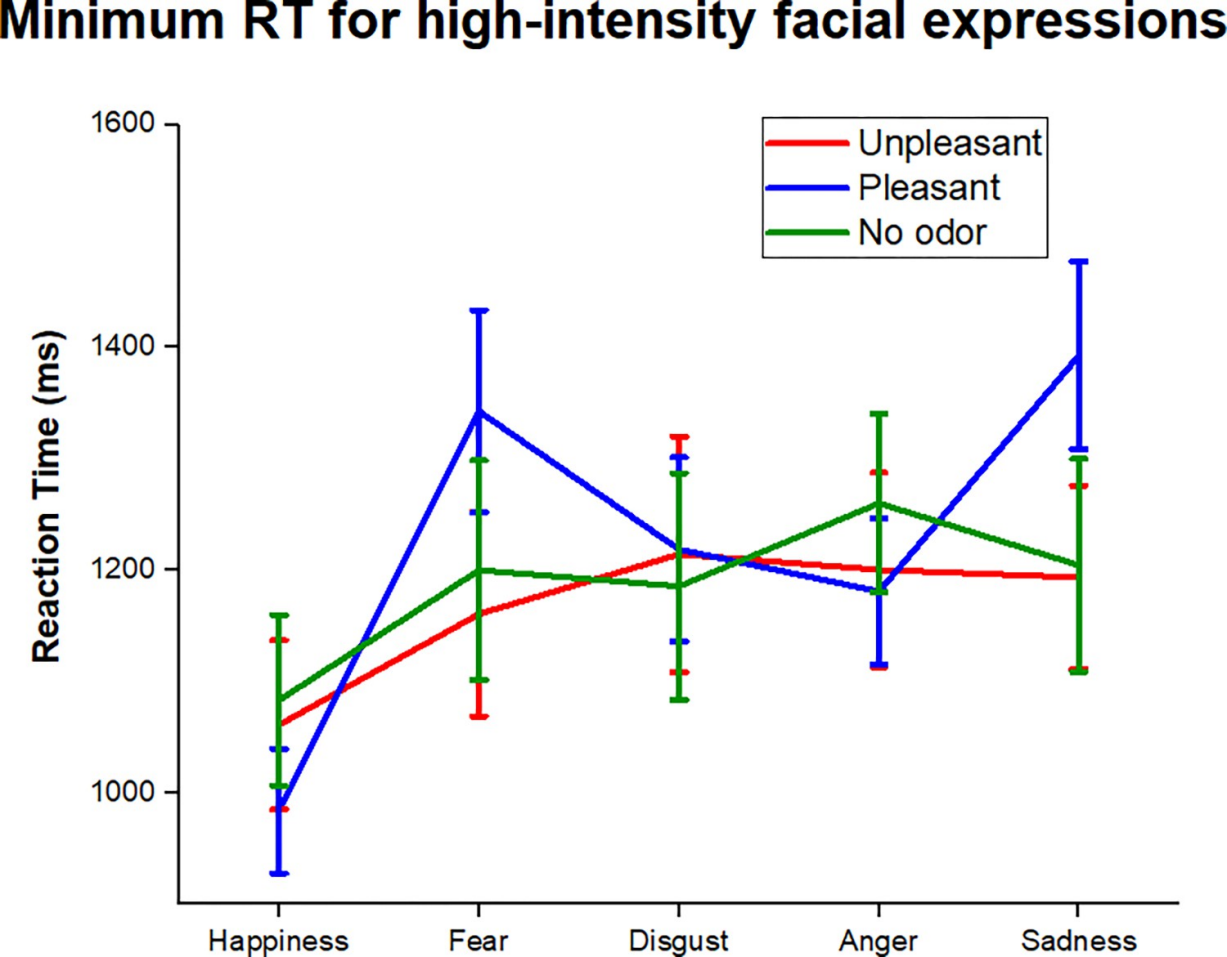
Difference in the minimum RT between emotions in each odor condition. Means and standard errors of the minimum RT for each emotion according to the odor condition.

A two-way ANOVA was conducted using Odor (unpleasant, pleasant and no odor) and Emotion (happiness, fear, disgust, anger and sadness) as within-subjects factors (Fig 7). Results show that the interaction between factors (Odor vs. Emotion) was significant (F = 2.26; p < .05; η_p_^2^ = .08). The Emotion factor also reached statistical significance (F = 4.13; p < .005; η_p_^2^ = .14), but the odor factor did not. The One-way ANOVAs for each odor condition confirmed that the Emotion factor was significant only for the pleasant condition (F = 6.82; p < .001; η_p_^2^ = .19), with happiness (983.7 ms) expressions presenting significantly lower RT than all emotions (all p < .05) except for disgust (1218.5 ms) (p > .05), but not in the neutral and unpleasant conditions. This means that the recognition speed superiority of happiness expressions compared to the other emotions were not present in the unpleasant condition. Although the emotion factor did not reach statistical significance in the no odor condition, pairwise comparisons showed a relevant difference between happiness (1083.1 ms) and anger (1260 ms) (p < .05).

## Discussion

Our study has found significant effects of olfactory stimulation in participants’ Reaction Time (RT) when completing a facial expressions identification task. More specifically, we showed that the odor stimuli have enhanced the RT differences between the intensities of fear and disgust facial expressions. Reaction Time decline following the increase of facial expression intensity varied depending on the odor condition presented. It is important to note that, since participants varied substantially in their threshold levels for each emotion category, we analyzed the RT of both correct and incorrect answers. Hence, our data is indicating the mean time subjects needed to complete the identification process in each odor condition and facial expression intensity. This means that the results address how odorant stimulation can modulate the emotional processing speed of facial expressions, and not the interference on the identification rate.

As we expected, the greatest differences of RT occurred between the threshold and medium intensity for all emotions in general, as full expressions results were more similar to the medium intensity of expressions. Leppänen. & Hietanen used low intensity facial expressions in order to increase the chances of participants emotional states interfere in the categorization of expression [12]. We believe that, after threshold intensity, the responses became more reliant in external (sensory) factors than internal emotional judgment. This is why, in our opinion, odorants probably influence differently the processing of facial expressions in these two distinct situations. There was a higher decrease in the RT mean of fear expressions between the threshold and medium intensities in the negative olfactory stimulation than other conditions. This pattern of results was repeated for other emotions, but in different olfactory conditions. In the case of disgust expressions, both the positive and negative odor stimuli presented a significant decline in RT after the threshold intensity that was not reproduced in the control condition [12]. Although it did not reach statistical significance, the happiness expressions showed the same pattern of results. Higher differences of RT means between intensities were found in the pleasant condition, compared to control and unpleasant. Compared to the control condition, unpleasant odors showed the same pattern for the RT of fear and disgust. At the threshold levels, it slowered responses, as if it was extending the stimuli processing or retarding responses. However, at higher intensities the unpleasant odor presented faster responses than other conditions, as if it was accelerating responses or the processing of the faces. The positive olfactory stimulation presented this pattern of results only for the disgust expressions, and reduced the descending curve of fear category as if it was expediting the responses for the threshold intensity. We believe that, in low intensities, odorants may have induced extended processing of stimuli in order to reach precision. At higher levels of expression the task is less dependent on internal processing, and congruency effects between olfactory stimuli and the facial expressions may act in order to accelerate responses since there is no necessity of higher processing (appraisal/judging).

Considering our hypothesis that odor effects on emotion recognition may be different depending on facial expressions intensity, we also analyzed the minimum Reaction Time needed to complete the task for high-intensity expressions. The happiness facial expressions were processed faster than other emotional categories, corroborating with previous experiments [12], but this effect also varied according to the olfactory condition presented. Compared to the no odor condition, the happiness superiority was lower in the presence of unpleasant olfactory stimulation and higher in the pleasant condition. Considering these results, we believe that unpleasant odors reduced the differences in RT between happiness and other emotions, but the pleasant stimuli enhanced the discrepancy. It is interesting to note that disgust and anger were the only emotions that did not differ from happiness in the pleasant condition. Somehow, this is in line with previous researches that did not find impairment in the perception of disgust during pleasant olfactory stimulation [12,19].

Similar to our results, Seubert et al. also found that both pleasant and unpleasant olfactory stimulation, independently of its hedonic valence, have enhanced the difference in subjects RT between expression intensities [19]. We suggest that, in a real world situation, the presence of an odor enhances the chances of people around to express facial expressions, considering human emotional reactivity in response to olfactory stimulation. Considering that disgust expressions were not influenced by the valence nature of olfactory stimuli, not just in our experiment, but also in Seubert et al. [19], we believe that a semantic element could play a role in this phenomenon. The pleasantness of an odorant is a subjective measure, as two people can have different emotional reactions to the same odor. Therefore, it is not unusual to see someone expressing a disgusted face while smelling an odorant that is considered as positively valenced by another person. That is why it may not be an advantage in social situations if the positive olfactory stimulation impairs the recognition of disgust facial expressions. Actually, the opposite would be a more logical explanation. In the presence of an odor, individuals may become more aware of emotional reactions that are congruent to the context of olfactory stimulation, which are happiness and disgust.

There may be other factors modelling the odor influences in emotion perception through facial expressions. In Cook et al. [9], happy and disgusted faces were rated as more pleasant in the positive olfactory stimulation, and less pleasant in negative odor condition. But the most important result of Cook’s experiment is that odorants were rated as more positive, in terms of hedonic valence, when presented with happiness expressions and as more intense in the case of disgust faces. This demonstrates a bilateral influence in the phenomenon, which highlights the importance of stimulus intensity selection, both visual and olfactory, and indicates that such aspect could also be one of the reasons for the contradictory results of previous studies. It was not just the set of odor stimuli that was not standardized, but also the visual stimuli set and its intensities (facial expressions). Different sets of faces probably differ in terms of the intensity of facial expressions, and possibly impact the emotional response to the odorants differently. As such, the differences in odorant and visual stimulation may be a contributing factor for the contradicting results in this field of research.

One example of the influence of stimulus selection for experiments involving odors was demonstrated by Pichon et al. [6]. In two experiments, these researchers used two groups of odorants to analyze emotional reactions, both subjective and physiological. The authors found more pronounced differences in physiological reactions between odors with higher hedonic contrast, although differences in subjective valence were present for both groups of odorants. The experiment that used a group of odorants (perfumes) with low valence contrast found no significant variations in physiological reactions between the odors that were rated subjectively with opposite valence. Therefore, for a group of olfactory stimuli with low valence contrast, subjects may evaluate an odor as unpleasant but may not present significant differences in physiological reactions compared with stimuli that are considered as pleasant. Future studies that use odors to elicit emotional reactions should consider promoting the highest hedonic contrast between stimuli.

The results from this research indicate that odorant effects can also highlight divergence in the processing time of facial expressions with different intensities. The presence of an odor enhanced the RT differences between threshold and higher facial expressions intensities, which could indicate a bilateral effect as described by Cook et al. [9]. The odorant influences the emotional response to the face but, considering the results of Cook et al. [9], the facial expressions can also elicit affective responses that influence the pleasantness rate of the olfactory stimulation. Therefore, compared to high-intensity facial expressions, threshold-intensity emotional faces may have a weaker influence on the affective reaction to the odorant. This means that, when a face is presented with a valence-congruent odorant (happy face and pleasant odor, for example), the facilitation effect may also depend on the intensity of the facial expression. If it is a low-intensity facial expression the affective categorization will possibly be slower and/or less effective, compared to a high-intensity one. The odorant stimulation in our study enhanced the RT differences between the facial expression intensities of fear and disgust. Nevertheless, high intensity facial expressions showed a different pattern of results, which may indicate that odorant influences may have varied between different facial expressions intensities. The bilateral hypothesis may explain why there is a significant divergence between the results of experiments in this field of research. In a recent review, Syrjänen et al discuss that individual differences in odor sensitivity related to age, gender or culture may also play a significant role in the phenomenon, and an experimental standardization is needed such as in the measures, testing procedures and also stimuli [20], which corroborates with our results. The differences in the affective impact of odorants and facial expressions, due to its intensity or intrinsic properties, between experiments in this area may influence the bilateral interplay between olfactory and visual emotional stimulation that is probably the key to this phenomenon.

In conclusion, although past studies have highlighted the role of hedonic valence in this phenomenon, there are several other factors that are possibly involved. Our results demonstrated that odors can induce an emotional response that may alter the emotional processing of faces, but this effect probably vary according to the intensity of the facial expression. This variation could be the result of a bilateral effect between the olfactory and visual stimuli, since the facial expressions can also interfere in the emotional processing of odorants [9]. Hence, the high-intensity facial expressions possibly attenuate or enhance the odorants’ emotional priming effect in the identification of emotions on faces. Future studies should be careful with methodological differences such as the visual and olfactory stimuli set and its emotional intensity, in order to standardize the method as much as possible to avoid such kinds of difference. The results from this research indicate that odor effects in the identification of faces are also modulated by the intensity of facial expressions, due to a bilateral emotional influence of both visual and olfactory stimulation. Our results also corroborate with the global perspective of the odor influence in facial expression recognition, since fear and sadness are emotions whose meaning are not in line with odor contexts.

## Supporting information

S1 Fig Analysis of RT between intensities for happiness, anger and sadness. Graphs showing RT means and standard errors for happiness, anger and sadness at threshold, medium and high expression intensities in different odor conditions.
(TIF)

S1 Table Reaction times, thresholds and odorant evaluation for each participant. Table showing individual data of the Reaction Times for each facial expression intensity, the thresholds for correct identification of each emotion and the subjective appraisals of odorants.
(XLSX)
