# Supplementary figures and images for: Olfactory interference on the emotional processing speed of visual stimuli: The influence of facial expressions intensities
Source: PLoS One. 2022 May 17;17(5):e0264261. doi: 10.1371/journal.pone.0264261 (PMC9113595; doi:10.1371/journal.pone.0264261)

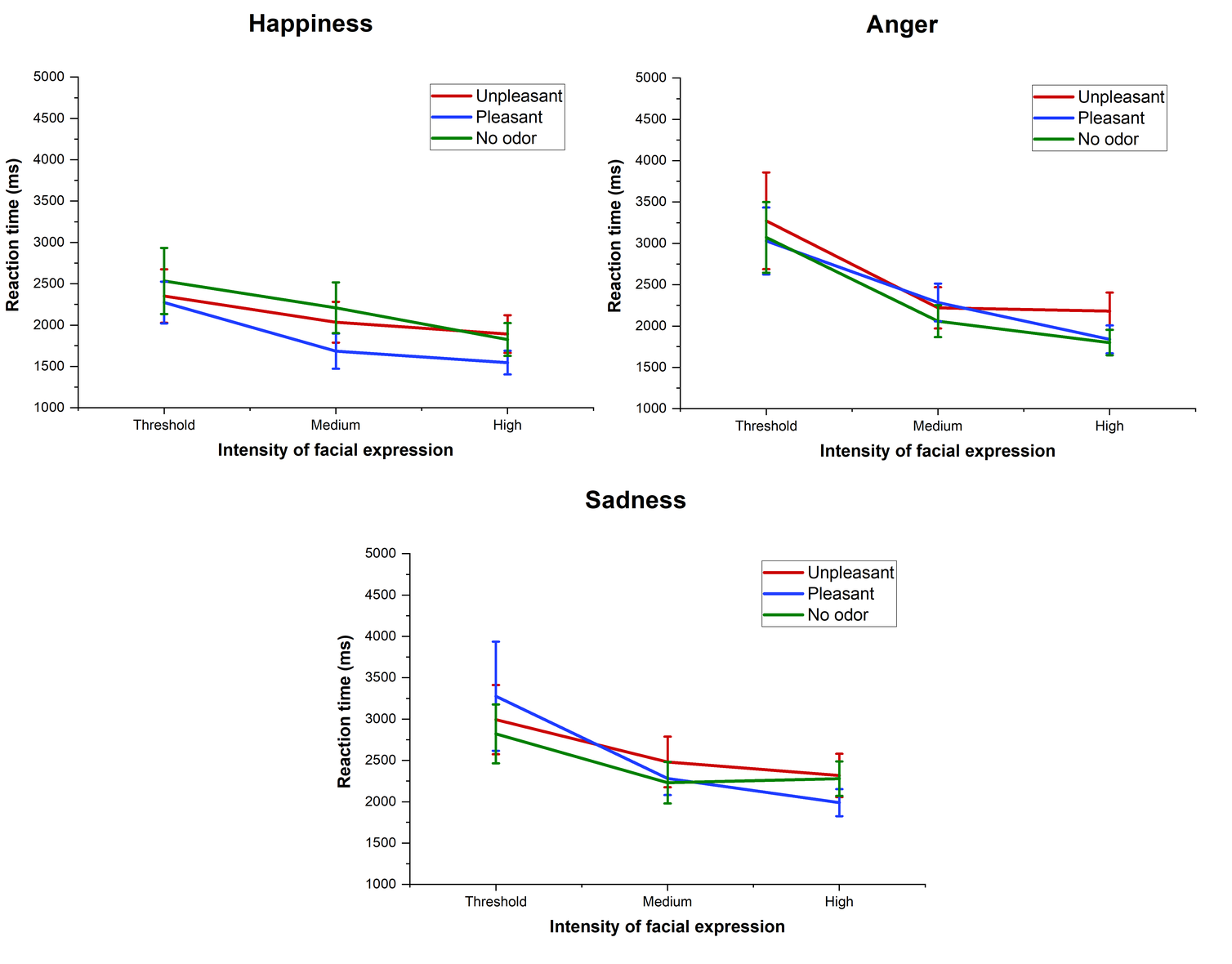

Supplement: S1 Fig — Graphs showing RT means and standard errors for happiness, anger and sadness at threshold, medium and high expression intensities in different odor conditions. (TIF) [file pone.0264261.s001.tif]
